# Studying the adsorption of emerging organic contaminants in zeolites with dispersion‐corrected density functional theory calculations: From numbers to recommendations

**DOI:** 10.1002/open.202300273

**Published:** 2024-02-22

**Authors:** Michael Fischer, Jakob Brauer

**Affiliations:** ^1^ Crystallography and Geomaterials Faculty of Geosciences University of Bremen Klagenfurter Straße 2–4 28359 Bremen Germany; ^2^ Bremen Center for Computational Materials Science and MAPEX Center for Materials and Processes University of Bremen 28359 Bremen Germany

**Keywords:** density functional theory, adsorption, zeolites, emerging organic contaminants, host-guest interactions, benchmarking

## Abstract

Adsorption energies obtained from dispersion‐corrected density functional theory (DFT) calculations show a considerable dependence on the choice of exchange‐correlation functional and dispersion correction. A number of investigations have employed different approaches to compute adsorption energies of small molecules in zeolites, using reference values from high‐level calculations and/or experiments. Such comparative studies are lacking for larger functional organic molecules such as pharmaceuticals or personal care products, despite their potential relevance for applications, *e. g*., in contaminant removal or drug delivery. The present study aims to fill this gap by comparing adsorption energies and, for selected cases, equilibrium structures of emerging organic contaminants adsorbed in MOR‐ and FAU‐type all‐silica zeolites. A total of 13 dispersion‐corrected DFT approaches are compared, including methods using a pairwise dispersion correction as well as non‐local van der Waals density functionals. While absolute values of adsorption energies vary widely, qualitative trends across the set of zeolite‐guest combinations are not strongly dependent on the choice of functional. For selected cluster models, DFT adsorption energies are compared to reference values from coupled cluster (DLPNO‐CCSD(T)) calculations. Although all DFT approaches deliver systematically more negative adsorption energies than the coupled cluster reference, this tendency is least pronounced for the rev‐vdW‐DF2 functional.

## Graphical abstract

The performance of 13 dispersion‐corrected density functional theory methods is compared systematically for adsorption energy calculations of emerging organic contaminants in all‐silica zeolites . While qualitative trends are similar, large differences in absolute values are observed. Among the tested approaches, the rev‐vdW‐DF2 functional reproduces reference values from high‐level wave function calculations most accurately.

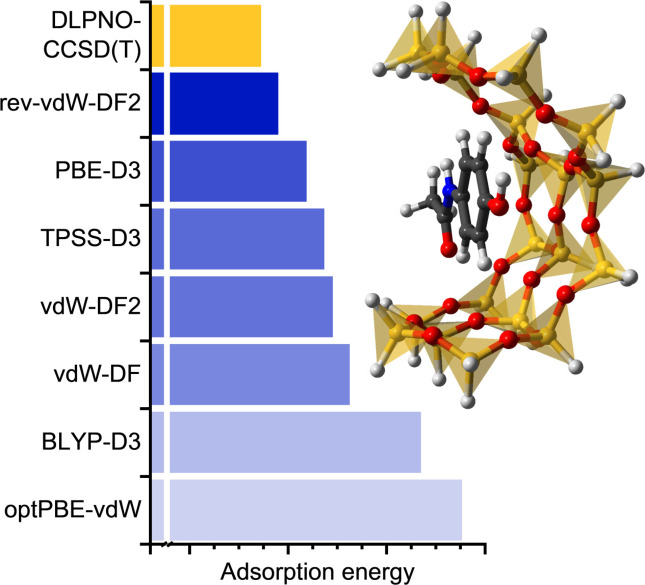

## Introduction

The adsorption of functional organic molecules, such as pharmaceuticals, personal care products, herbicides, insecticides, etc., in high‐silica and all‐silica zeolites is relevant in the context of different applications: On the one hand, these hydrophobic adsorbents could be employed in the removal of emerging organic contaminants from wastewaters.^[1]^ Promising results have been obtained, for example, for the use of faujasite‐type zeolite Y (FAU framework^[2]^) to remove sulfonamide antibiotics,[^3^, ^4^] other drugs like carbamazepine,^[5]^ and the personal care product triclosan,^[6]^ for high‐silica mordenite (MOR framework) as adsorbent for various pharmaceuticals and other organic contaminants,[^7^, ^8^] and for zeolite beta (BEA framework) in the removal of drugs,^[9]^ pesticides,^[10]^ and perfluoroalkyl substances.[^11^, ^12^] On the other hand, high/all‐silica zeolites could also find use as carrier materials in the controlled delivery of drug molecules[^13^, ^14^, ^15^, ^16^] or for the encapsulation of organic UV filters, enhancing their UV filtering activity while at the same time preventing undesired release into the environment.^[17]^ Further potential applications of zeolites involving the adsorption of sizeable organic molecules from the liquid phase include the recovery of valuable fractions from complex mixtures, *e. g*., in the processing of rapeseed,^[18]^ and the use of zeolites as solid sorbents in (micro)extraction.^[19]^

Given the vast number of potentially interesting organic species, and the multitude of zeolite frameworks that are available in highly or purely siliceous form (about 70 zeolite frameworks have been synthesised as all‐silica zeolites to date[^2^, ^20^]), it is clear that experimental investigations of the adsorption properties are feasible only for a small fraction of the theoretically possible adsorbent‐guest combinations. To narrow down the selection, atomistic simulations can be employed to gauge the affinity of different zeolites towards a species of interest. Recent work showed a good correlation between zeolite‐guest interaction energies and experimentally observed removal efficiencies for 21 organic contaminants in MOR‐ and FAU‐type zeolites.^[21]^ Interaction energies were computed using a fairly simplistic approach based on force field (FF) Monte Carlo (MC) simulations and subsequent energy minimisations with the DREIDING FF,^[22]^ indicating that even relatively crude simulations could be very useful to identify adsorbent‐guest combinations of interest. Other authors have employed FF‐based molecular dynamics (MD) simulations to study the diffusion of pharmaceuticals through zeolite structures in the context of drug delivery investigations.[^14^, ^15^, ^16^]

A crucial aspect in this regard is the suitability of the FF parameters to represent the relevant interatomic interactions in the system, especially the interactions between zeolite host and adsorbed molecules. For gas phase adsorption, a validation against experimental adsorption data is relatively straightforward, and high‐quality FFs that accurately reproduce adsorption isotherms for gases like CO_2_, CH_4_, N_2_, or O_2_ in all‐silica zeolites have been developed.[^23^, ^24^, ^25^, ^26^] For larger organics that are adsorbed from the liquid phase, the situation is more complex, as a direct comparison to liquid‐phase adsorption isotherms not only requires a reasonably accurate description of the interactions between zeolite and organic guest, but also of interactions between the organic species and the solvent (typically water). Some progress in this direction has been made, and expanded‐ensemble MC simulations have been shown to reproduce liquid‐phase adsorption data rather well.[^27^, ^28^] Altogether, however, a validation against experimental data remains a very complex endeavour, not least due to the impact of structural defects (usually ignored in the simulations) on the experimental isotherms.

An alternative approach is the validation of FF parameters against electronic structure calculations, which can also be employed for cases where no experimental adsorption data are (yet) available. Moreover, an analysis of the results obtained at a higher level of theory can provide insights into the nature of the host‐guest interactions. Given its good scaling behaviour and efficient implementation for periodic systems, density functional theory (DFT) is the most widely applicable electronic structure method for these systems. Due to the well‐known shortcomings of standard DFT in describing long‐range dispersion interactions, which play an important role for adsorption phenomena, recent investigations have usually employed dispersion‐corrected “flavours” of DFT. For example, the DREIDING calculations carried out in one of the aforementioned studies^[21]^ were supplemented by DFT calculations for selected systems, which used the Perdew‐Burke‐Ernzerhof (PBE)^[29]^ functional with a D3 dispersion correction.^[30]^ It was shown that FF and DFT give, by and large, the same trends, validating the conclusions drawn from the DREIDING calculations. On the basis of preliminary results obtained in the context of the present study, the rev‐vdW‐DF2 functional, which includes a non‐local dispersion correction,^[31]^ was used in recent DFT studies of carbamazepine and triclosan adsorption in all‐silica and high‐silica zeolites.[^32^, ^33^] Schwalbe‐Koda and Goméz‐Bombarelli compared binding energies obtained from static and dynamic DREIDING calculations to reference values from DFT optimisations and DFT‐based MD simulations using the PBE‐D3 functional, considering a total of 227 combinations of zeolites and organic structure‐directing agents (OSDAs).^[34]^ Pointing out that the PBE‐D3 functional tends to overbind guest molecules (see below), they noted that it provides a good balance between accuracy and computational cost compared to more sophisticated approaches. Altogether, they observed a good correlation between FF and DFT calculations, especially when using the “frozen‐pose” method to compute the DREIDING binding energies (calculation of the host‐guest binding energy without relaxation of zeolite and OSDA). They proposed this method for large‐scale FF‐based screening studies of zeolite‐OSDA pairs.

It is well established that DFT interaction energies show a strong dependence on the choice of exchange‐correlation (XC) functional, which governs the description of short‐range interactions, and (if used) the method to include long‐range dispersion interactions. As a large variety of XC functionals and dispersion correction schemes are available, there is a plethora of possible combinations. Several authors have compared different approaches by calculating adsorption energies of small molecules (hydrocarbons, alcohols, CO_2_, H_2_O) in all‐silica zeolites,[^35^, ^36^, ^37^, ^38^, ^39^] as well as protonated and cation‐exchanged zeolites,[^23^, ^36^, ^37^, ^39^, ^40^, ^41^, ^42^, ^43^] using values extrapolated from experiment and/or results of higher‐level calculations as benchmarks. Without going into the details of the individual studies, the following common findings can be summarised:

- XC functionals using the generalised gradient approximation (GGA) without any dispersion correction severely underestimate adsorption energies because the dispersion contribution is missing.
- For alkanes, standard GGA functionals like the Perdew‐Burke‐Ernzerhof (PBE)^[29]^ functional in conjunction with pairwise dispersion corrections, like the D2^[44]^ or D3 corrections^[30]^ developed by Grimme and co‐workers or the TS correction proposed by Tkatchenko and Scheffler,^[45]^ have a systematic tendency to overestimate the interaction strength, *i. e*., they deliver “too negative” adsorption energies.[^36^, ^37^, ^38^, ^40^, ^41^, ^42^] While the tendency is generally observed, the degree of overestimation varies across studies. It also depends on the specific adsorbate studied.
- The tendency to overbind is typically more pronounced when using different flavours of non‐local van der Waals density functionals (vdW‐DF).[^36^, ^37^, ^38^, ^40^, ^41^, ^43^]
- Similar trends are observed for other molecules like acetylene, ethylene, and CO_2_.[^23^, ^42^] It is, however, also worth noting that acceptable agreement of PBE‐D2 adsorption energies with experimental values was observed for methanol, ethanol, and propanol in MFI‐type zeolites,^[37]^ and that revPBE‐D3 performed very well for H_2_O adsorption energies in CHA‐type zeolites.^[39]^

Studies comparing adsorption energies of functional organic molecules in zeolites are rarer, and typically include a smaller number of DFT approaches.[^46^, ^47^, ^48^, ^49^] In this context, it has to be emphasised that the validation of DFT calculations dealing with larger molecules faces additional challenges: On the one hand, comparisons to experimental adsorption enthalpies (where available) are much less straightforward for molecules adsorbed from the liquid phase as compared to gases. On the other hand, higher‐level reference calculations require larger model systems (supercells of zeolite unit cells to avoid overlap of a molecule with its images or large clusters cut out from the zeolite structure), quickly becoming computationally very demanding.

While coupled cluster with singles, doubles, and perturbative triples (CCSD(T)) calculations are commonly considered as the “gold standard” of quantum chemistry,[^50^, ^51^] their computational cost is so high that the zeolite framework can only be represented using a small cluster model consisting of a few tetrahedrally coordinated atoms (T atoms). CCSD(T) calculations for such small clusters have been used as starting point to benchmark computationally less demanding methods, such as dispersion‐corrected DFT or the random phase approximation (RPA).[^42^, ^52^, ^53^, ^54^] Correcting DFT results with pre‐established correction factors based on coupled‐cluster calculations can result in accurate energies (DFT/CC method[^23^, ^38^, ^55^]). However, the calculation of these correction factors incurs a significant computational overhead for previously unstudied adsorbates. Embedding approaches in which different parts of the system are treated at different levels of theory (QM:QM methods) can deliver highly accurate adsorption energies,[^43^, ^56^, ^57^, ^58^] but may be difficult to apply for large guest molecules, where a significant fraction of the zeolite framework would have to be treated at the highest level of theory.

The domain based local pair‐natural orbital CCSD method in conjunction with an improved perturbative triples correction, dubbed DLPNO‐CCSD(T),^[59]^ can deliver results of canonical CCSD(T) quality at a significantly reduced computational cost.^[60]^ Recently, this method has found several uses in the zeolite field, both for the calculation of reference energies for different guest molecules interacting with zeolite clusters models, including protonated and cation‐exchanged systems,[^47^, ^48^, ^61^, ^62^] and to compute correction terms to energies obtained at a lower level of theory.[^63^, ^64^, ^65^, ^66^]

So far, no systematic comparisons of DFT adsorption energies obtained with different approaches have been reported for emerging organic contaminants like pharmaceuticals or personal care products. The present study aims to fill this gap, reporting results obtained with a total of 13 dispersion‐corrected exchange‐correlation (dc‐XC) functionals, including five GGA‐type functionals and one meta‐GGA functional in conjunction with a pairwise D3 correction as well as seven vdW‐DF methods. For a few adsorption complexes, a validation against DLPNO‐CCSD(T) calculations is carried out in the last part, using suitably sized cluster models. This investigation is structured as follows:

In **Part 1**, adsorption energies are computed for a variety of organic species in two all‐silica zeolites (MOR and FAU), with the choice of adsorbents and adsorbates following previous experimental and computational work.[^7^, ^21^] In addition to evaluating the differences in absolute adsorption energies, it is also assessed whether different functionals deliver any appreciable differences in the relative trends.

**Part 2** concentrates on three molecules (acetaminophen – ACA, ibuprofen – IBU, triclosan – TCS) in MOR. For each molecule, different adsorption complexes are considered in order to test to what extent the energetic ordering of different configurations of the same molecule in the same zeolite depends on the chosen DFT approach.

Finally, **Part 3** compares DFT results to adsorption energies from DLPNO‐CCSD(T) calculations. Due to the computational expense of the coupled cluster calculations, periodic zeolite models are replaced by cluster models in this part, and only the smallest guest molecule, acetaminophen, is considered.

It should be emphasised that this work does not attempt to obtain DFT‐computed adsorption energies that could be directly compared to any observable quantity. Instead, the key aim is to identify trends within the set of dc‐XC functionals, and to benchmark them against higher‐level calculations, at least for a selection of models. On this basis, some recommendations regarding their probable suitability can be made, allowing to use them with confidence in future, more practically oriented studies dealing with the adsorption of emerging organic contaminants or similarly complex organic molecules in zeolites.

## Computational Details

### Details of DFT calculations

All calculations used the Quickstep electronic structure module within the CP2K code (**Part 1, 2**: version 7.1, **Part 3**: version 9.1), which uses a Gaussian and plane wave approach^[67]^. For a given organic species *Org* in a zeolite, the adsorption energy was calculated as:

(1) $$E_{ads}=\;E_{DFT,opti}\left(Zeo+Org\right)-E_{DFT,sp}\left(Zeo\right)-E_{DFT,opti,box}\left(Org\right)$$

Here, the first term on the right‐hand side corresponds to the DFT total energy obtained from a structure optimisation of *Org* adsorbed in the pores of the zeolite (adsorption complex), the second term is the single‐point energy of the guest‐free zeolite framework, and the third term corresponds to the energy of the optimised, isolated contaminant molecule. The positions of framework atoms were held fixed in all structure optimisations, although it is clear that local relaxations of the zeolite framework upon guest molecule adsorption should be taken into account in a comprehensive DFT treatment. In the context of the present work, however, it was deemed preferable to restrict the comparison to host‐guest interaction energies and (for selected cases) the positions of adsorbed molecules. Apart from increasing the computational cost, including a relaxation of the zeolite framework would add an additional layer of complexity.^[47]^ A considerable dependence of the optimised structural parameters (bond lengths, angles, cell parameters) on the chosen DFT approach has been observed in comparative studies of guest‐free zeolites.[^68^, ^69^, ^70^, ^71^] It can hence be anticipated that they would also predict a different degree of adsorption‐induced deformation, rendering it more difficult to isolate the contribution of host‐guest interactions in a comparative analysis. Several prior benchmarking studies of host‐guest interactions in zeolites kept the atomic coordinates of the framework atoms fixed.[^35^, ^42^, ^48^]

The DFT calculations in **Part 1** and **2** employed molecularly optimised (MOLOPT) basis sets from the work of VandeVondele and Hutter.^[72]^ Both double‐zeta short‐range basis sets (DZVP‐MOLOPT‐SR labelled “DZVP‐SR” for brevity in the following) and triple‐zeta basis sets (TZVP‐MOLOPT) were used as discussed in the Results section. Preliminary tests showed that the use of larger triple‐zeta basis sets (TZV2P‐MOLOPT and TZV2PX‐MOLOPT) resulted in only marginal changes in the adsorption energies that usually remained below 3 %. Only the Γ point was used to sample the first Brillouin zone. The plane wave energy cutoff was set to 600 Ry as preliminary calculations indicated that this value gave well converged adsorption energies (use of an energy cutoff of 800 Ry typically resulted in changes by less than 2 %). Goedeker‐Teter‐Hutter pseudo‐potentials developed by Krack were used to represent the core electrons.^[73]^ Structure optimisations were considered converged when the following convergence criteria were simultaneously met: Maximal residual force below 2.5 ⋅ 10^−6^ Ha bohr^‐1^ and maximal displacement below 5 ⋅ 10^−5^ bohr. For calculations on isolated contaminant molecules the periodicity was switched off (keyword: PERIODIC NONE). A wavelet solver was used to solve Poisson's equation for these non‐periodic systems.^[74]^ In calculations with non‐local vdW‐DF functionals it was found that a Fast Fourier Transform (FFT) cutoff of 400 Ry gave converged results.

### Dispersion‐corrected DFT approaches

A total of 13 dc‐XC functionals were compared, using otherwise identical settings. They are listed in Table 1, which also groups conceptually similar functionals together. While the reader is referred to the references in Table 1 for details on individual functionals, and to exhaustive review and benchmarking articles for further information,[^75^, ^76^, ^77^, ^78^, ^79^] these groups are very briefly described in the following:

**Table 1:** Overview of dispersion‐corrected DFT approaches used in this work.

Label	Group	References	Label	Group	References
PBE‐D3	GGA+D3	[29, 30]	vdW‐DF	Non‐local vdW‐DF1	[82, 83]
revPBE‐D3	GGA+D3	[30, 83]	vdW‐DF‐cx	Non‐local vdW‐DF1	[82, 87]
BLYP‐D3	GGA+D3	[30, 88, 89]	vdW‐DF‐C09	Non‐local vdW‐DF1	[82, 90]
BP86‐D3	GGA+D3	[30, 85, 88]	optPBE‐vdW	Non‐local vdW‐DF1	[82, 91]
B97‐D3	GGA+D3	[30, 44, 92]	optB88‐vdW	Non‐local vdW‐DF1	[82, 91]
TPSS‐D3	meta‐GGA+D3	[30, 80]	vdW‐DF2	Non‐local vdW‐DF2	[84, 86]
			rev‐vdW‐DF2	Non‐local vdW‐DF2	[31, 84, 85]

1. The five GGA+D3 functionals combine different GGA functionals with the pairwise D3 dispersion correction developed by Grimme and co‐workers.^[30]^ GGA‐type functionals make use of the electron density and its gradient to compute the XC contribution to the total energy. In the calculations, the originally proposed “zero damping” of the dispersion term was used, and the coefficients to scale the D3 dispersion correction depend on the XC functional (see https://www.chemiebn.uni‐bonn.de/pctc/mulliken‐center/software/dft‐d3/functionals).
2. TPSS‐D3 is the only meta‐GGA functional combined with the D3 dispersion correction considered in the present work.^[80]^ Meta‐GGA functionals also employ information on the kinetic energy density to compute the XC contribution, they are computationally moderately more expensive than GGA functionals.
3. The “van der Waals density functional” (vdW‐DF) approach proposed by Dion et al. combines GGA exchange and LDA correlation^[81]^ with a non‐local correlation contribution, which depends on the electron density and a non‐local kernel.^[82]^ It is thus designed to incorporate dispersion interactions in a seamless fashion, obviating the need for element‐specific dispersion coefficients. As systematic inaccuracies were identified in the original vdW‐DF approach, which uses revPBE exchange,^[83]^ several combinations with other exchange functionals have been proposed subsequently. In addition to the original implementation, four of these “Non‐local vdW‐DF1” approaches were considered in this study (Table 1 **)**.
4. To improve upon some shortcomings of the vdW‐DF1 approach, especially overestimated equilibrium distances and exaggerated attractive dispersion interactions, a modified version of the non‐local kernel was implemented by Lee et al. in their vdW‐DF2 method.^[84]^ A further improved version of vdW‐DF2, which uses a revised B86b exchange functional^[85]^ instead of PW86 exchange,^[86]^ was later proposed by Hamada (rev‐vdW‐DF2).^[31]^

### DLPNO‐CCSD(T) and DFT calculations for cluster models

In **Part 3**, single‐point DLPNO‐CCSD(T) calculations were carried out to investigate the interaction of acetaminophen, the smallest organic contaminant molecule considered, with cluster models extracted from the structures of MOR and FAU, taking PBE‐D3 optimised structures as starting points, as described in more detail below. For these calculations, the ORCA program package, version 5.0.4, was used.^[93]^ All calculations employed the “def2” basis sets developed by the Ahlrichs group,^[94]^ using split‐valence/double zeta up to quadruple zeta basis sets. Auxiliary basis sets for Coulomb^[95]^ and exchange^[96]^ fitting and for correlation^[97]^ were used. Single point energies obtained with the ORCA code were extrapolated to the complete basis set (CBS) limit using the implemented extrapolation method by Neese and Valeev.^[98]^ DLPNO‐CCSD(T)[^59^, ^60^] calculations were run using tight self‐consistent field and tight pair natural orbital settings in line with the recommendations for the investigation of weak non‐covalent interactions.

To ensure comparability of between results obtained with ORCA and CP2K, adsorption energies were also computed on the DFT level using selected dc‐XC functionals that are implemented in the ORCA code (PBE‐/revPBE‐/BLYP‐/BP‐/TPSS‐D3 functionals, see Table 1). To attain consistency with the CP2K calculations, the D3 method with zero damping was used.

DFT calculations on non‐periodic clusters were also performed using CP2K, using the wavelet Poisson solver.^[74]^ The calculations used identical cutoff values as the periodic DFT calculations above, but employed different types of basis sets: In order to allow for an extrapolation to the complete basis set limit, as done in the ORCA calculations, triple‐zeta and quadruple‐zeta basis sets were included, using TZVP‐GTH, TZV2P‐GTH, QZV2P‐GTH, and QZV3P‐GTH basis sets from the GTH_BASIS_SETS file included in the CP2K distribution. To allow for better comparability with other parts of the study, calculations on clusters were also done with TZVP‐MOLOPT‐GTH basis sets, but these results were not included in the basis set extrapolation. The extrapolation to the CBS limit used a cubic extrapolation formula:

(2) $$E_{DFT,X}=E_{DFT,X\to \infty }+AX^{-3}$$

Here, X is the cardinal number of the basis set and $E_{DFT,X\to \infty }$ corresponds to the total energy in the CBS limit. This energy is obtained as intercept of a linear fit with the slope A . The cardinal number X corresponds to 3 for TVZP‐/TZV2P‐GTH basis sets and 4 for QZV2P‐/QZV3P‐GTH basis sets. The cubic extrapolation formula was originally proposed for an extrapolation of the correlation energy part in post‐Hartree Fock calculations,^[99]^ but was subsequently also applied to total energies.[^100^, ^101^] Although tailored variants of the extrapolation formula have been proposed, *e. g*., for use with specific types of basis sets, they tend to give similar results as equation 2 if the larger basis set is of quadruple‐zeta or higher quality.[^102^, ^103^]

### Models of organic contaminants, zeolites, and adsorption complexes

Molecular structures of 21 organic contaminants were taken from earlier work.^[21]^ Table 2 lists these contaminants, including sum formula, molecular weight, and typical applications. The structures had been obtained from the PubChem^[104]^ and CheBi^[105]^ databases, and were pre‐optimised with the DREIDING force field.^[22]^ As mentioned above, non‐periodic calculations were run for these molecules to avoid artificial interactions between molecules in adjacent simulation cells. Likewise, the structure models of all‐silica MOR and FAU were the same as in earlier work.^[21]^ These models were taken from the IZA Database^[2]^ and optimised using the force field of Sanders, Leslie, and Catlow (SLC),^[106]^ which has been shown to give good agreement with experimental structure data for all‐silica zeolites[^69^, ^107^, ^108^] Whereas the regular unit cell was used for FAU, a 1×1×3 supercell was employed for MOR. For the calculations performed in **Part 1**, the initial structures of the adsorption complexes of 15 contaminants in MOR and 21 contaminants in FAU were taken from the previous force field study,^[21]^ in which the choice of contaminants had been based on earlier experimental work dealing with MOR‐ and FAU‐type adsorbents.^[7]^ Whereas the previous study considered 21 contaminants in both MOR and FAU, six species for which the Monte Carlo insertion into the pores of MOR either failed entirely or was at least problematic were not included in the present work (see Table 2). These omissions were motivated by the intention to take a set of adsorption complexes obtained via a consistent computational approach as starting point. In **Part 2**, additional adsorption complexes of ACA, IBU, and TCS adsorbed in MOR were investigated. These configurations were generated via an MD‐based annealing approach, using the Forcite module of the DS BIOVIA “Material Studio” suite.^[109]^ For each system, two annealing runs, each consisting of 25 heating‐cooling cycles within a temperature interval between 300 and 1000 K, were performed, employing the DREIDING force field.^[22]^ At the end of each heating‐cooling cycle, the structures were optimised with the same force field. For ACA/IBU/TCS, 2/4/4 adsorption complexes were selected from the annealing results. These were studied together with the configurations that were already included in **Part 1**, so a total of 3/5/5 configurations were considered.

**Table 2:** List of organic contaminants considered in this study. Abbreviations are specified only for those species where the abbreviation is either used in the text or frequently encountered in the literature. The sixth column specifies whether the species were included in Part 1 for MOR and FAU (M+F) or FAU only (F).

	Abbr.	Sum formula	*m*_molar_ [g mol^−1^]	Application	Part 1	Part 2
Acetaminophen	ACA	C_8_H_9_NO_2_	151.17	Analgesic	M+F	M
Atrazine		C_8_H_14_ClN_5_	215.69	Herbicide	M+F	
Caffeine		C_8_H_10_N_4_O_2_	194.19	Stimulant	M+F	
Carbamazepine	CBZ	C_15_H_12_N_2_O	236.27	Anticonvulsant	F	
*N*,*N*‐diethyl‐*meta*‐toluamide	DEET	C_12_H_17_NO	191.27	Insect repellent	M+F	
Diazepam		C_16_H_13_ClN_2_O	284.75	Anti‐anxiety agent	F	
Diclofenac		C_14_H_11_Cl_2_NO_2_	296.15	NSAID	F	
Dilantin (Phenytoin)		C_15_H_12_N_2_O_2_	252.27	Anticonvulsant	F	
Estrone		C_18_H_22_O_2_	270.37	Estrogen	M+F	
Fluoxetine	FLX	C_17_H_18_F_3_NO	309.33	Antidepressant	M+F	
Gemfibrozil		C_15_H_22_O_3_	250.34	Lipid regulator	M+F	
Hydrocodone		C_18_H_21_NO_3_	299.37	Analgesic	F	
Ibuprofen	IBU	C_13_H_18_O_2_	206.29	NSAID	M+F	M
Meprobamate		C_9_H_18_N_2_O_4_	218.25	Anti‐anxiety agent	M+F	
Naproxen		C_14_H_14_O_3_	230.26	NSAID	M+F	
Oxybenzone		C_14_H_12_O_3_	228.25	UV absorber	M+F	
Pentoxifylline		C_13_H_18_N_4_O_3_	278.31	Rheological agent	F	
Sulfamethoxazole		C_10_H_11_N_3_O_3_S	253.28	Antibiotic	M+F	
Tri(2‐chloroethyl) phosphate	TCEP	C_6_H_12_Cl_3_O_4_P	285.49	Flame retardant	M+F	
Triclosan	TCS	C_12_H_7_Cl_3_O_2_	289.55	Antibacterial agent	M+F	M
Trimethoprim		C_14_H_18_N_4_O_3_	290.32	Antibiotic	M+F	

NSAID=Non‐steroidal anti‐inflammatory drug.

Because CCSD(T) calculations are not feasible for periodic structures, cluster models representing the most significant part of the zeolite framework were employed in **Part 3**. These cluster models were extracted from the adsorption complexes optimised with the PBE‐D3 functional, considering two different configurations for MOR and one for FAU. The closest part of the zeolite framework was cut out from the periodic structure, resulting in MOR models with 19 T atoms and a FAU model with 18 T atoms. Dangling oxygen atoms were replaced by hydrogen atoms, fixing all Si–H distances to 1.48 Å.^[110]^

## Results

### Part 1: Adsorption of various contaminants in MOR and FAU

The structures of the adsorption complexes (15 species in MOR, 21 species in FAU) and of isolated contaminants were optimised using DZVP‐SR basis sets, and the computed adsorption energies are collected in Table S1.1 (EXCEL spreadsheet **S1**). The adsorption energies were then recomputed using TZVP‐MOLOPT basis sets, employing the DZVP‐SR‐optimised structures (Table S1.2). A comparison of the $E_{ads}$ values obtained with the different basis sets reveals appreciable differences, with the DZVP‐SR basis sets always delivering larger absolute values (= more negative adsorption energies). Interestingly, the effect of the basis set size varies rather markedly across the set of 13 functionals: For four functionals (BLYP‐D3, vdW‐DF, optPBE‐vdW, vdW‐DF2), the average relative deviation, computed over 36 individual values, is on the order of −11 to −12 %, whereas it amounts to −19 to −22 % for several other functionals including PBE‐D3, TPSS‐D3, vdW‐DF‐C09, and rev‐vdW‐DF2. Due to the important influence of the basis set size, the following discussion considers exclusively the adsorption energies obtained with the larger TZVP‐MOLOPT basis sets.

**Figure 1:**
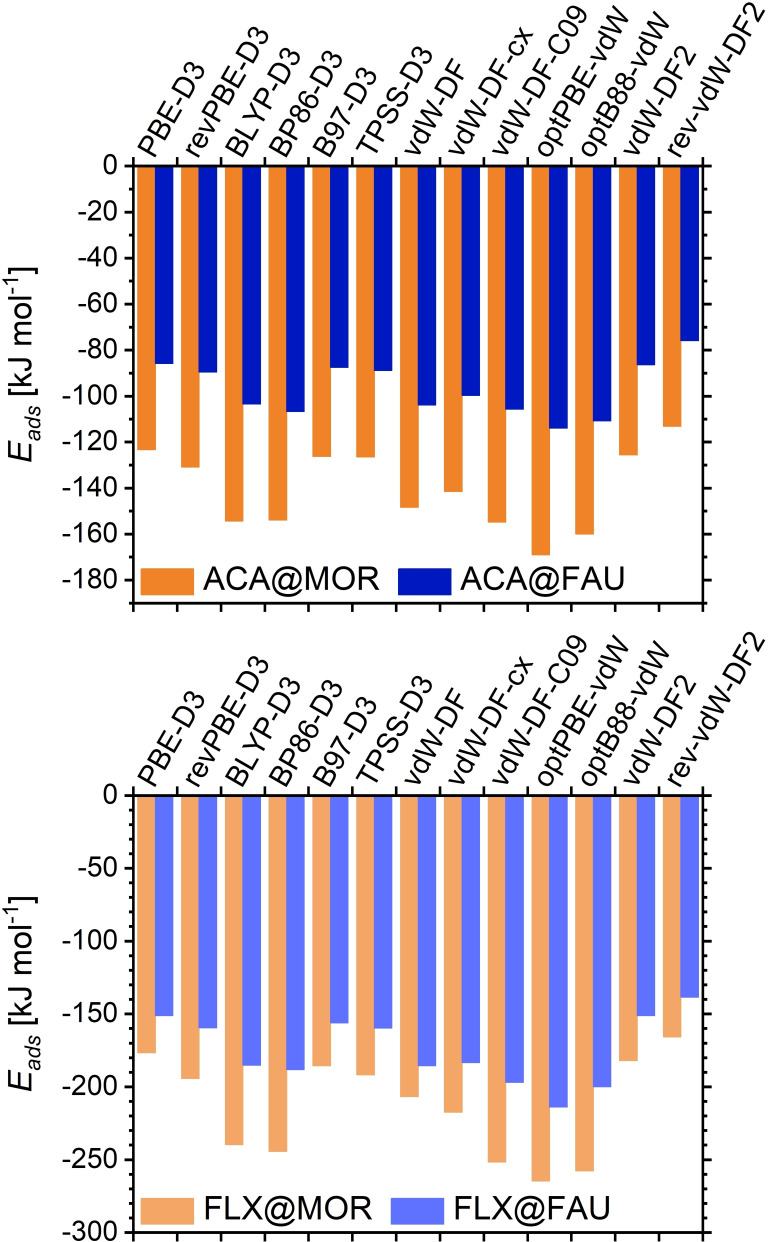
Adsorption energies computed for ACA (top) and FLX (bottom) in MOR (orange columns) and FAU (blue columns) using different dc‐XC functionals.

When comparing the adsorption energies computed for a given molecule in one zeolite, the large dependence on the choice of dc‐XC functional is immediately apparent. This is illustrated for the smallest and largest contaminant species, acetaminophen (ACA) and fluoxetine (FLX), in Figure 1, but a similar overall picture would arise for every other contaminant. Starting with ACA@MOR, the most negative adsorption energy value is obtained using the optPBE‐vdW functional, with −169 kJ mol^−1^, whereas rev‐vdW‐DF2 delivers the least negative value of −113 kJ mol^−1^. For ACA@FAU, the same two functionals give the most/least negative adsorption energies of −114 kJ mol^−1^ and −76 kJ mol^−1^. The interaction in FAU is significantly weaker than in MOR due to the larger pore size, and hence fewer framework atoms in the distance range of strongest dispersion interactions, as discussed in earlier work.^[21]^ For FLX, which interacts much more strongly due to its larger size, optPBE‐vdW also results in the most negative adsorption energies, which amount to −265 kJ mol^−1^ and −214 kJ mol^−1^ for adsorption in MOR and FAU, respectively. In contrast, the rev‐vdW‐DF2 functional predicts adsorption energies of −166 kJ mol^−1^ and −139 kJ mol^−1^. Across the set of energy values, optPBE‐vdW delivers the strongest interaction for all but three zeolite‐contaminant combinations, where optB88‐vdW and in one case also BP86‐D3 give more negative values. On the other side of the spectrum, rev‐vdW‐DF2 gives the least negative $E_{ads}$ values for all but three combinations, where different functionals using the D3 dispersion correction (PBE‐D3, revPBE‐D3, B97‐D3, TPSS‐D3) result in less negative values. The differences between the maximal and minimal $E_{ads}$ values for a given zeolite‐guest combination fall between 33 and 99 kJ mol^−1^, or between 30 and 40 % in relative terms.

**Figure 2:**
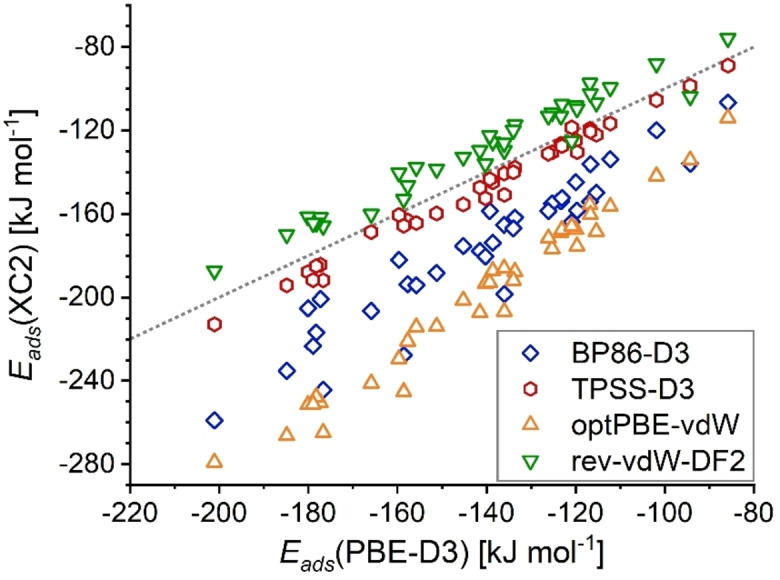
Adsorption energies for the whole set of contaminants adsorbed in MOR and FAU, computed with different dc‐XC functionals and plotted against the PBE‐D3 adsorption energies. The dotted line indicates a slope of 1.

On the basis of the results presented above, it can be clearly stated that the choice of dc‐XC functional has a very large impact on the absolute values of the adsorption energies. However, there will be situations where it is more relevant to identify qualitative trends reliably, rather than having a means to compute accurate absolute values. Such qualitative trends could be important, for example, in a search for zeolite adsorbents having a particularly strong affinity towards a given contaminant. It is therefore important to assess to what extent the trends predicted by different functionals differ. For illustrative purposes, the adsorption energies obtained with the BP86‐D3, TPSS‐D3, optPBE‐vdW, and rev‐vdW‐DF2 functionals are plotted against the PBE‐D3 energies in Figure 2. It has to be emphasised that the use of PBE‐D3 as reference is made for convenience here, and is not meant to imply that the PBE‐D3 energies are expected to be of superior accuracy. In principle, any other functional could be taken as reference.

Figure 2 shows essentially linear trends for all four functionals, with only a few outliers that appear to be more prominent for BP86‐D3 and rev‐vdW‐DF2 compared to TPSS‐D3 and optPBE‐vdW. Whereas the adsorption energies obtained with TPSS‐D3 and rev‐vdW‐DF2 fall slightly below and above the grey line that represents a perfect linear correlation with a slope of 1, BP86‐D3 and especially optPBE‐vdW deliver systematically more negative adsorption energies, as could be expected from the discussion above. For a more quantitative assessment, it is useful to calculate least‐square linear regressions of the form $E_{ads}\left(XC2\right)={m_{12}\cdot E}_{ads}\left(XC1\right)$ , where XC1=PBE‐D3 and XC2=another dc‐XC functional (the intercept is set to zero because no offset should occur in the limit of no interaction). For the four functionals, the following slopes and squared correlation coefficients $R^{2}$ were obtained: BP86‐D3: $m_{12}$ =1.252, $R^{2}$ =0.892; TPSS‐D3: $m_{12}$ =1.047, $R^{2}$ =0.987; optPBE‐vdW: $m_{12}$ =1.413, $R^{2}$ =0.973; rev‐vdW‐DF2: $m_{12}$ =0.918,$\;R^{2}$ =0.955.

**Figure 3:**
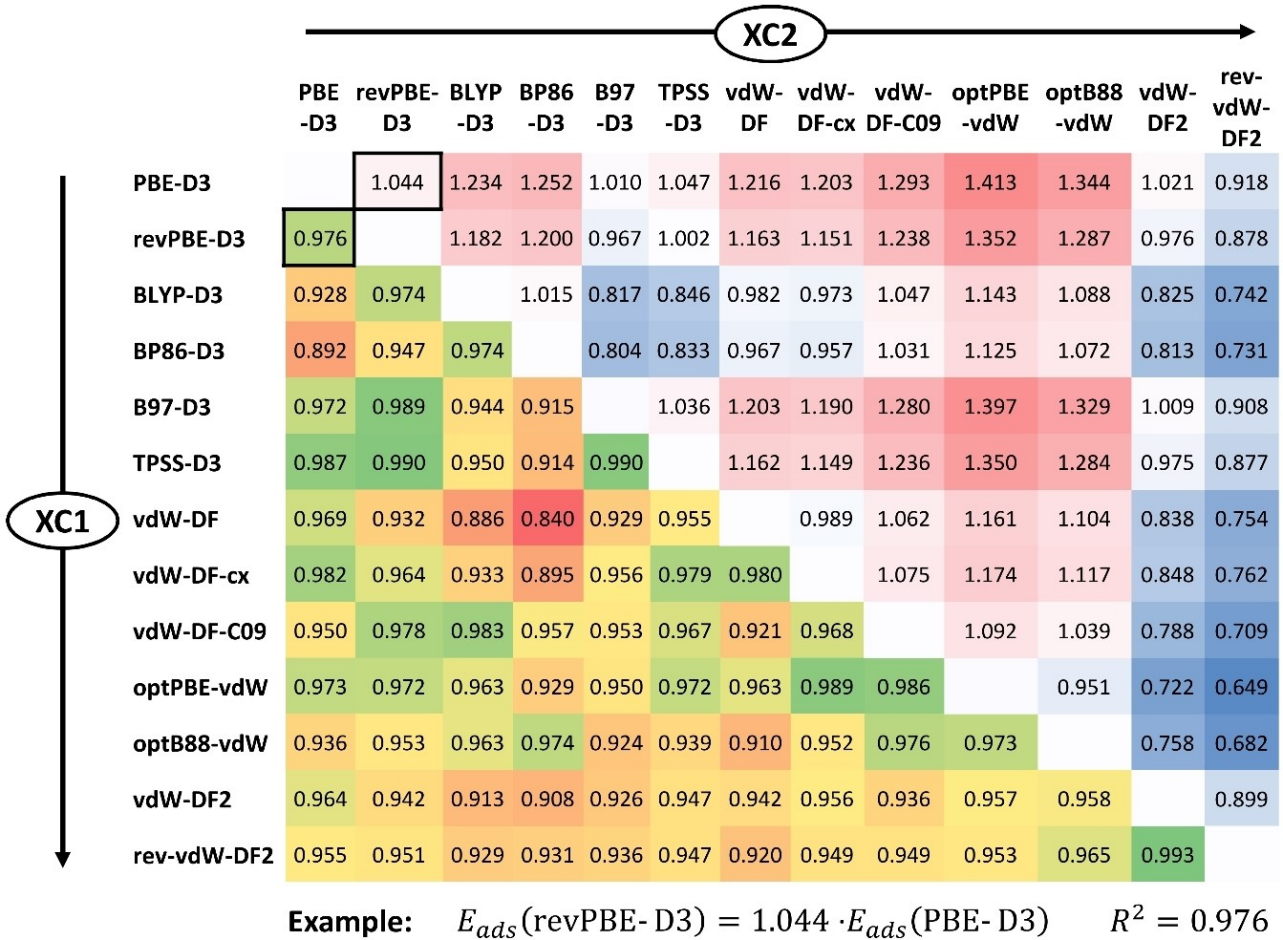
**Top right**: Slopes $m_{12}$ of linear regressions obtained on the basis of adsorption energies computed with exchange‐correlation functionals XC1 and XC2. Increasingly blue/red background indicates slopes smaller/larger than 1. **Bottom left**: Squared correlation coefficients $R^{2}$ for combinations of functionals. The values for the example XC1=PBE‐D3, XC2=revPBE‐D3 are highlighted with black frames. Green/red background indicates more/less strong correlation (yellow/orange=intermediate).

In order to evaluate the similarities and differences among the functionals in a more comprehensive fashion, regression lines across the dataset were computed, using all combinations of XC1 and XC2. The results are collected in Figure 3, where the top right half reports the slopes of the linear regression lines and the bottom left half gives the respective squared correlation coefficients. Redundant slope values that could be computed as $m_{21}=1/m_{12}$ (with the same $R^{2}$ ) are omitted. On the basis of these results, the XC functionals can be grouped as follows:

- Three of the five GGA+D3 functionals, PBE‐/revPBE‐/B97‐D3, as well as the meta‐GGA TPSS‐D3 functional give adsorption energies of very similar magnitude ($m_{12}$ values ranging from 0.97 to 1.05) and the results are highly correlated with each other ($R^{2}$ >0.97).
- The other two GGA+D3 functionals, BLYP‐D3 and BP86‐D3, deliver adsorption energies that are, on average, 23 to 25 % more negative than those obtained using PBE‐D3. While the results obtained with these two functionals are highly correlated, correlations with other functionals are less prominent, with most $R^{2}$ values being smaller than 0.95.
- Adsorption energies that are 20 to 40 % more negative than the PBE‐D3 energies are obtained with the functionals belonging to the vdW‐DF1 group. They exhibit a varying degree of correlation with each other and with other functionals.
- vdW‐DF2 gives rather similar results as PBE‐D3, with $m_{12}$ amounting to 1.02 and a squared correlation coefficient of 0.96. rev‐vdW‐DF2 is the only functional for which the slope $m_{12}$ for XC1=PBE‐D3 is smaller than one, with the adsorption energies being about 8 % less negative. Apart from a very strong correlation with vdW‐DF2, correlations with other functionals fall in a range of 0.93<$R^{2}$ <0.97.

Altogether, the results from this part show that use of different functionals results in pronounced differences in the magnitude of absolute adsorption energies. On the other hand, all functionals deliver very similar qualitative trends, with even the smallest squared correlation coefficient amounting to 0.84, and all except four $R^{2}$ values being larger than 0.90.

#### Part 2: Different adsorption configurations of ACA, IBU, and TCS in MOR

**Figure 4:**
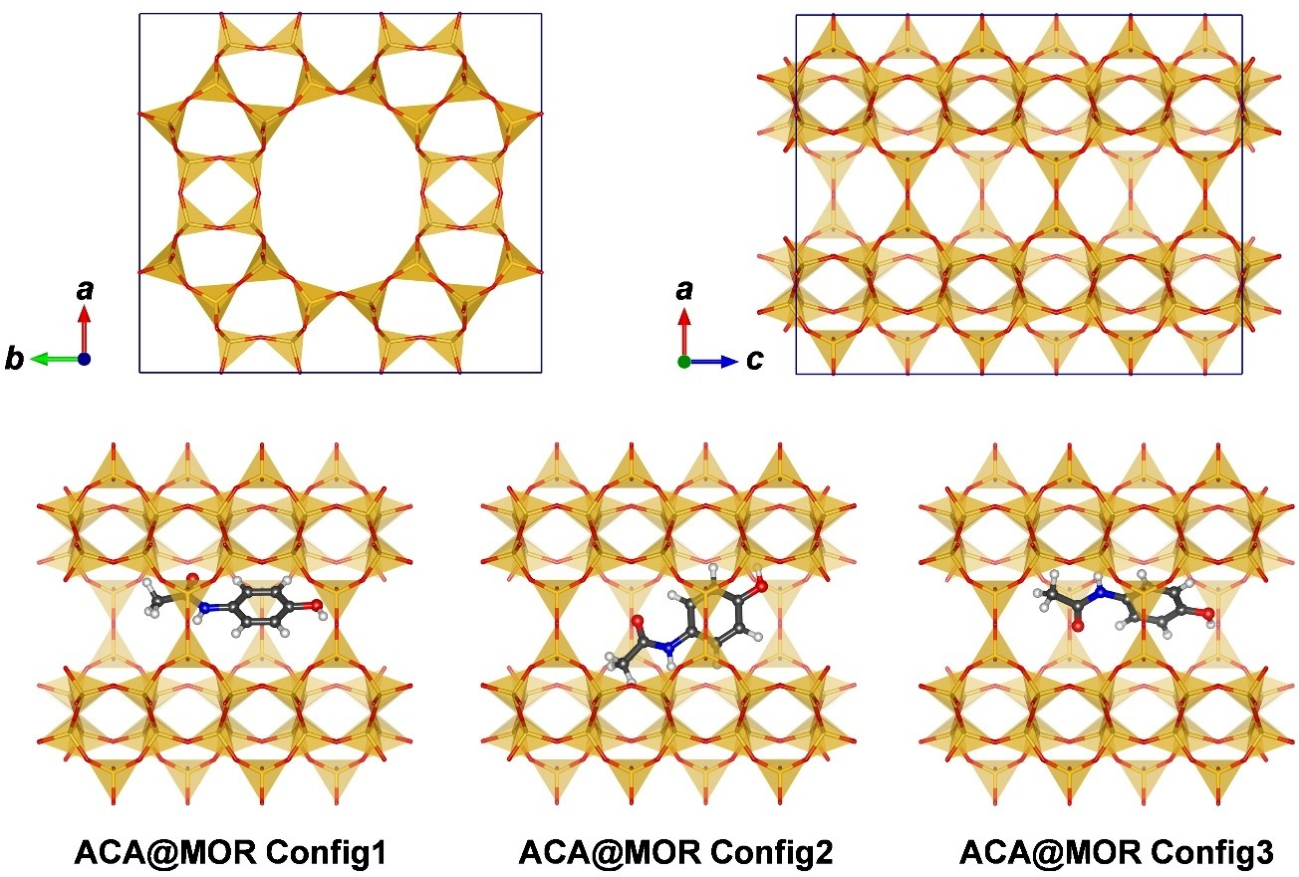
**Top**: Visualisation of the unit cell of MOR in projections along [001] and [010]. The 12‐membered ring channels run along the *c* direction. **Bottom**: Adsorption configurations of acetaminophen in the channels of MOR. Only the surrounding portion of the channel is shown.

Having shown the large impact of the choice of functional on the total adsorption energy, it is now interesting to assess to what extent the energetic ordering of different configurations of the same molecule in one zeolite is affected, both in terms of adsorption energies and structures of the adsorption complexes. For this purpose, the investigation focussed on three organic contaminants, acetaminophen, ibuprofen, and triclosan, which differ considerably in terms of adsorption energies and conformational degrees of freedom. Figure 4 shows the adsorption configurations of ACA@MOR that were included in the calculations (IBU@MOR and TCS@MOR configurations are shown in Table S2.0 of EXCEL spreadsheet S2). In each case, Config1 corresponds to the configuration that was already included in **Part 1**, and the other configurations (2/4/4 for ACA/IBU/TCS) were generated using preliminary annealing simulations with the DREIDING force field.

Unlike in **Part 1**, the structures of the adsorption complexes (and of free contaminant molecules) were optimised using TZVP‐MOLOPT basis sets. For the Config1 cases, these adsorption energies can be directly compared to adsorption energies obtained from TZVP‐MOLOPT single‐point calculations on DZVP‐MOLOPT‐SR‐optimised structures (“TZVP‐MOLOPT/DZVP‐SR”). These results are collected in the lower part of Table S2.1. The differences between TZVP‐MOLOPT/TZVP‐MOLOPT and TZVP‐MOLOPT/DZVP‐SR adsorption energies remain below 5 kJ mol^−1^ (in absolute terms), with the mean of absolute errors across the three contaminants not exceeding 2.3 kJ mol^−1^, and remaining below 1.0 kJ mol^−1^ for 8 out of 13 functionals. As the total adsorption energies fall between −110 and −250 kJ mol^−1^, such changes can be considered negligible.

**Figure 5:**
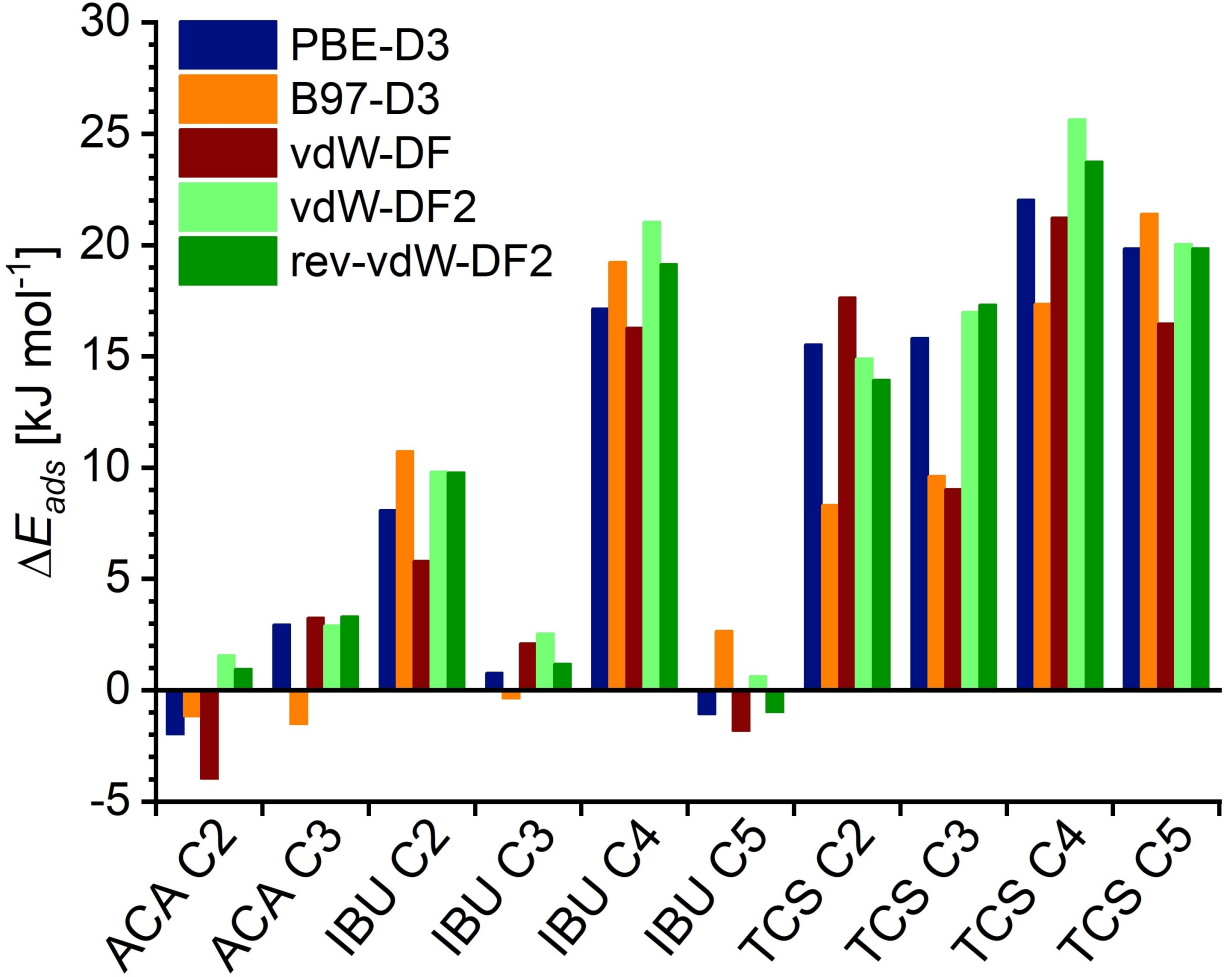
Relative energy ${\Delta E}_{ads}$ for different configurations of ACA/IBU/TCS@MOR

Table S2.1 also contains the adsorption energies obtained for all 13 configurations with the different dc‐XC functionals. An analysis of correlations analogous to that of **Part 1** gives similar results to those discussed above (Table S2.2), since it is dominated by the absolute adsorption energy values. In order to assess the energetic ordering of different configurations, the energy differences ${\Delta E}_{ads}$ with respect to the Config1 models (relative energies) were calculated (Table S2.3). Figure 5 shows the ${\Delta E}_{ads}$ values obtained with the PBE‐D3, B97‐D3, vdW‐DF, vdW‐DF2, and rev‐vdW‐DF2 functionals. For ACA@MOR Config2, IBU@MOR Config3, and IBU@MOR Config5, all functionals give ${\Delta E}_{ads}$ values close to zero, but the sign differs, in other words, different functionals disagree on whether the configurations are slightly more or less stable than the respective Config1. Even though a similarly small ${\Delta E}_{ads}$ value is calculated for ACA@MOR Config3 (in other words, all three ACA@MOR configurations are very close together in energy), very consistent results are obtained with all shown functionals except B97‐D3. For the remaining configurations, where the ${\Delta E}_{ads}$ values are significantly larger, PBE‐D3, vdW‐DF2, and rev‐vdW‐DF2 deliver results within a few kJ mol^−1^ of each other, as well as giving the same energetic orderings (*e. g*., for TCS@MOR: Config1 – Config2 – Config3 – Config5 – Config4). B97‐D3 and vdW‐DF both show larger absolute deviations, as well as predicting a different ordering of the configurations.

While the absolute values obtained with different functionals fall in similar ranges, the relative deviations in individual values may be considerable, especially for small ${\Delta E}_{ads}$ values. Hence, the usefulness of a linear fit through the origin is limited. On the other hand, an analysis of correlations between ${\Delta E}_{ads}$ values can still be insightful. The resulting $R^{2}$ values are compiled in Table S2.4. Among the (meta−)GGA+D3 functionals, PBE‐D3, revPBE‐D3, and TPSS‐D3 give fairly highly correlated results. Interestingly, the ${\Delta E}_{ads}$ values obtained with three functionals from the vdW‐DF1 group (vdW‐DF‐C09, optPBE‐vdW, optB88‐vdW) and with vdW‐DF2 and rev‐vdW‐DF2 are highly correlated with the PBE‐/revPBE‐/TPSS‐D3 results, and with each other. A less prominent, but still significant correlation is observed for vdW‐DF and vdW‐DF‐cx. In contrast, ${\Delta E}_{ads}$ values computed with the BLYP‐D3, BP86‐D3, and B97‐D3 functionals are relatively poorly correlated with those calculated with other functionals.

Looking beyond adsorption energies, it is also useful to analyse to what extent the choice of functional affects the equilibrium structure of the adsorbed contaminant (as noted above, structural parameters of the zeolite framework were held fixed in all calculations). While a detailed analysis could make use of various intermolecular quantities (bond lengths, bond angles, dihedral angles) as well as distances to framework atoms, it would quickly become rather cumbersome, given the number of configurations and dc‐XC functionals considered. As it is the main aim of the present analysis to determine how similar or different the configurations obtained with different functionals are, a less refined approach was used: In this approach, the PBE‐D3 optimised structures were taken as reference, and the distance vectors $\vec{v_{i}}$ between the positions of a given atom i in the structure optimised with another functional and its position in the PBE‐D3 structure were computed. The average positional deviation dev was then calculated over all N atoms as $dev=\frac{1}{N}\sum_{i}^{N}\left|\vec{v_{i}}\right|$ .^[111]^ As in **Part 1**, the use of PBE‐D3 coordinates as reference is arbitrary, as coordinates optimised with any other functional could equally well be used. However, PBE‐D3 appears as an appropriate choice because numerous other dc‐XC functionals give relatively similar ${\Delta E}_{ads}$ values.

**Figure 6:**
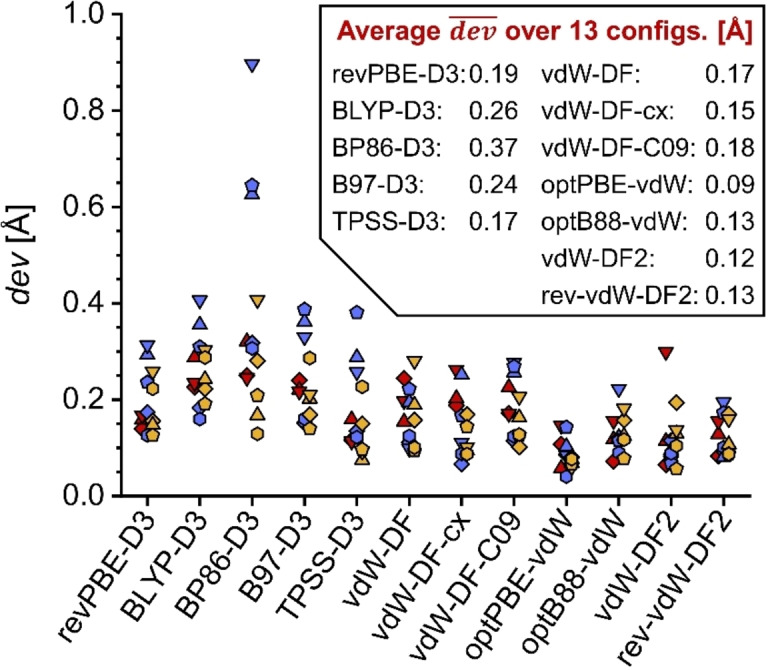
Average deviation dev in atomic coordinates of adsorbed ACA/IBU/TCS molecules with respect to PBE‐D3 reference coordinates. Individual deviations are represented with symbols, using the following scheme: ACA@MOR=red symbols, IBU@MOR=blue symbols, TCS@MOR=yellow symbols. Config1=diamonds, Config2=upright triangles, Config3=inverted triangles, Config4=pentagons, Config5=hexagons. To avoid symbol overlap, symbols for ACA and TCS are slightly shifted to the left and right, respectively. Averages over all configurations are given in the inset.

The deviations for individual configurations are shown in Figure 6 (coordinates are compiled in Tables S2.6 to S2.8, and numerical values of dev are given in Table S2.9). The average $\bar{dev}$ values calculated over all 13 configurations are also included in that figure. Closest agreement with the PBE‐D3 structures is found for optPBE‐vdW, followed by vdW‐DF2, rev‐vdW‐DF2, and optB88‐vdW. For all these functionals, most individual dev values remain below 0.15 Å, indicating only relatively modest differences in the positions of the adsorbed molecules. This agrees with the correlation in the ${\Delta E}_{ads}$ values found above. The remaining three vdW‐DF1 functionals form a second tier, where a significant number of individual configurations have dev values above 0.2 Å, but below 0.3 Å, indicating reasonable similarity with the PBE‐D3 structures. Despite using the same dispersion correction as PBE‐D3, the other GGA+D3 functionals and TPSS‐D3 result in at least one individual configuration having a dev value above 0.3 Å. Among these, the overall deviation from the PBE‐D3 structures is less pronounced for revPBE‐D3 and TPSS‐D3, which also deliver relatively similar ${\Delta E}_{ads}$ values. The other three functionals give very different equilibrium structures than PBE‐D3 in several instances, with the most pronounced differences occurring for some IBU@MOR configurations optimised with the BP86‐D3 functional.

All three molecules considered in this part contain OH groups. Hydrogen bonds between these groups and framework oxygen atoms O_fw_ may make a significant contribution to the host‐guest interaction. It is therefore interesting to evaluate to what extent different functionals differ in the description of these bonds in terms of hydrogen bond distances and angles. To determine whether hydrogen bonds are present or not, the configurations optimised with the PBE‐D3 functional were analysed, considering all cases as hydrogen‐bonded where the *d*(H_OH_⋅⋅⋅O_fw_) distance is smaller than 2.5 Å. On this basis, hydrogen bonds were found in 7 out of 13 configurations: ACA@MOR Config2 and 3, IBU@MOR Config1 and 5, TCS@MOR Config2, 3, and 4 (visualised in Table S2.10). Even though there are a few instances where functionals other than PBE‐D3 give distances slightly below 2.5 Å for additional configurations, the use of the PBE‐D3 structures as reference point is consistent with the approach used above for the overall deviations in coordinates. In almost all cases, the shortest *d*(H_OH_⋅⋅⋅O_fw_) contacts are formed to the same framework oxygen atom in configurations optimised with different functionals, with the few exceptions corresponding to configurations having rather large $\bar{dev}$ values (*e. g*., TCS@MOR Config3 optimised with BP86‐D3 and BLYP‐D3). When looking at the individual *d*(H_OH_⋅⋅⋅O_fw_) distances, listed in Table S2.11, some scatter is evident, with the difference between the shortest and longest distance obtained with different functionals varying between 0.13 and 0.31 Å. However, there are no clearly discernible trends in the sense that some functionals deliver systematically longer distances than others. As a consequence, a calculation of the average over all seven configurations results in relatively similar values, which fall in a range from 2.31 to 2.38 Å. Corresponding observations can be made for the hydrogen bond angles.

As pointed out previously, the positions of the framework atoms were held fixed in all calculations. It can be anticipated that an additional relaxation would affect the results, both with regard to the energetic ordering of different configurations and the equilibrium positions of adsorbed molecules. Especially for oxygen atoms that act as hydrogen bond acceptors, it seems plausible to expect certain changes in the Si−O_fw_−Si angles, which may result in somewhat shorter and stronger hydrogen bonds compared to calculations that do not relax the zeolite framework. While this might in turn change the energetic ordering of individual configurations, it appears unlikely that the observed similarities and differences across the set of functionals would be altered.

#### Part 3: Comparison of DFT and DLPNO‐CCSD(T) calculations for cluster models

**Figure 7:**
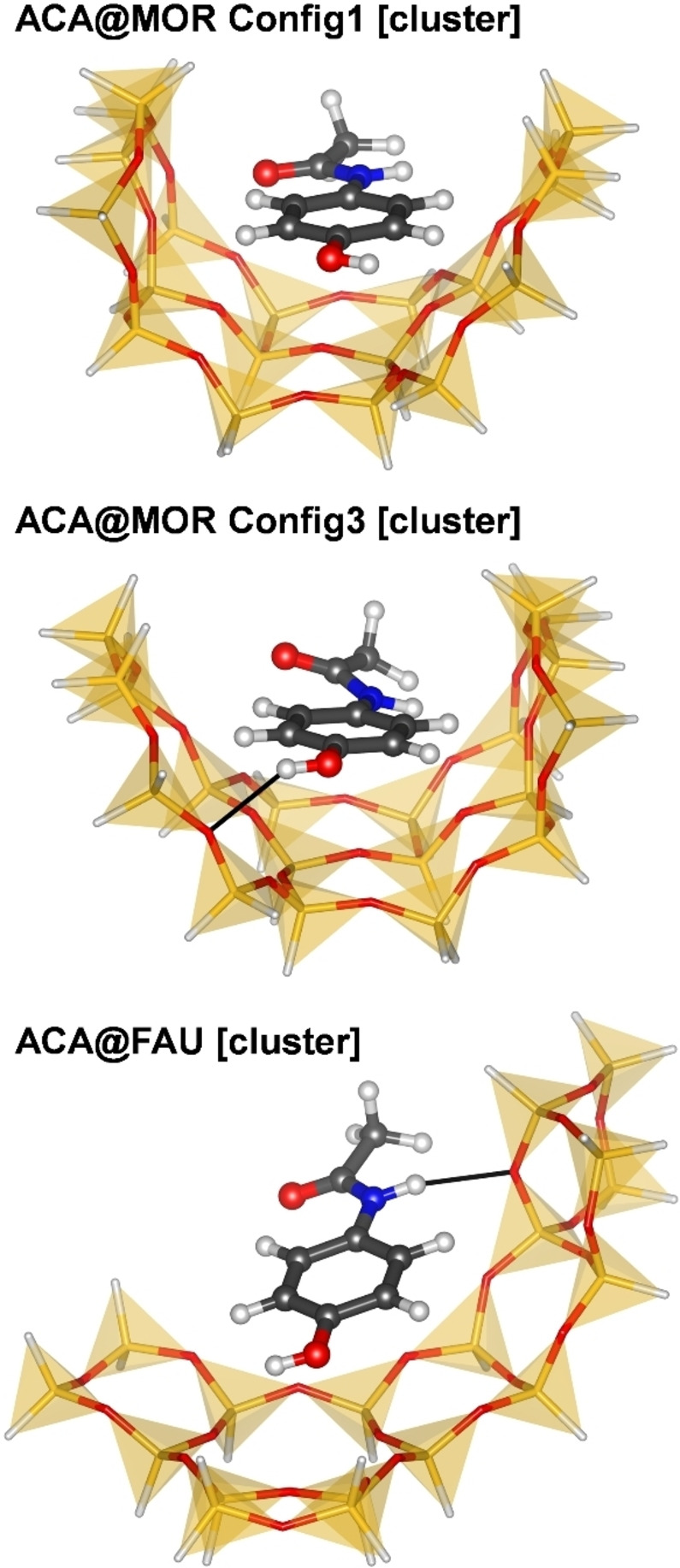
Cluster models used in calculations of **Part 3**. Thin black lines indicate hydrogen bonds.

Due to the infeasibility of periodic DLPNO‐CCSD(T) calculations, these calculations were performed for two cluster models of ACA@MOR adsorption complexes studied in **Part 2**, as well as a cluster model of the ACA@FAU complex studied in **Part 1**. For ACA@MOR, Config1 and Config3 were chosen for the following reasons: First, the ACA molecules are displaced from the channel centre, interacting primarily with one side of the channel wall. Therefore, a relatively small cluster model should be able to capture the largest part of the host‐guest interactions. In contrast, the ACA molecule in Config2 lies across the channel, interacting with both sides, so a much larger model would be required (Figure 4). Second, although DFT calculations deliver very similar adsorption energies for both configurations, Config3 contains a H_OH_⋅⋅⋅O_fw_ hydrogen bond, whereas Config1 does not. All cluster models were cut out from the structures optimised using the PBE‐D3 functional and TZVP‐MOLOPT (for MOR) or DZVP‐SR (for FAU) basis sets. They are visualised in Figure 7.

Because full structure optimisations in the framework of the DLPNO‐CCSD(T) method are not feasible at present, only single‐point calculations were carried out. To evaluate all methods on an equal footing, the DFT adsorption energies were computed for the same cluster models, cut out from periodic structures in which the position of acetaminophen was optimised using the PBE‐D3 functional (dubbed “frozen” clusters in the following because no further optimisations of the clusters were performed). In order to test whether the trends in adsorption energies across the set of functionals that were established in **Part 1** are affected by the use of such frozen cluster models, rather than periodic models optimised with the dc‐XC functional of interest, the adsorption energies were recomputed for the cluster models. These calculations compared TZVP‐MOLOPT basis sets to triple‐ and quadruple‐zeta GTH basis sets, the latter of which also allowed for a CBS extrapolation. The results are summarised in Table S3.1. The adsorption energies obtained for clusters are significantly less negative, which is not surprising given the missing contribution of long‐range dispersion interactions. When calculating a linear regression between adsorption energies obtained from calculations for frozen clusters and those obtained from periodic calculations, the squared correlation coefficients are≥0.90 for individual GTH or MOLOPT basis sets, and fall between 0.86 and 0.91 when using CBS extrapolation for the cluster models. It is worth noting that the correlation coefficients increase to values of≥0.93 if only a single functional, namely the vdW‐DF functional, is omitted, and the correlation improves even further if vdW‐DF‐cx is also omitted (with all $R^{2}$ values being≥0.96). At this point, it remains unclear why a larger difference between periodic and cluster calculations occurs for these two functionals. By and large, however, the analysis convincingly shows that the trends among the different functionals observed previously for periodic structure optimisations are retained, validating the outlined strategy of using single‐point calculations on clusters cut out from the PBE‐D3 optimised structures in the benchmarking of DFT approaches against DLPNO‐CCSD(T) calculations.

Since the reference DLPNO‐CCSD(T) calculations were performed with ORCA, using the Ahlrichs (denoted “def2”) basis sets, it was evaluated in the next step whether adsorption energies obtained with such calculations are directly comparable to those of CP2K calculations with GTH basis sets. Since only some of the dc‐XC functionals listed in Table 1 are implemented in ORCA, five (meta−)GGA+D3 functionals were included in this comparison: PBE‐D3, revPBE‐D3, BLYP‐D3, BP86‐D3, and TPSS‐D3. The results are compiled in Table S3.2, which also includes the mean of absolute error MAE of CP2K versus ORCA adsorption energies for comparable basis set sizes. When employing TZV2P‐GTH and QZV3P‐GTH basis sets in CP2K, the MAE values obtained for different functionals are on the order of 1.2 to 2.3 kJ mol^‐1^ and 2.1 to 3.5 kJ mol^‐1^, respectively (reference: ORCA def2‐TZVPP and def2‐QZVPP adsorption energies). Interestingly, CP2K results obtained with TZVP‐MOLOPT basis sets fall more or less in the middle between the ORCA def2‐TZVPP and def2‐QZVPP adsorption energies. The most impressive agreement, however, is achieved by extrapolating to the CBS limit: CBS extrapolation of CP2K TZV2P‐/ QZV3P‐GTH energies delivers MAE values that do not exceed 1.1 kJ mol^−1^ when CBS‐extrapolated ORCA def2‐TZVPP/def2‐QZVPP results are taken as reference. It can thus be inferred that CBS extrapolation of TZV2P‐/QZV3P‐GTH energies in CP2K delivers $E_{ads}$ values that can be directly benchmarked against (also CBS‐extrapolated) DLPNO‐CCSD(T) values calculated with ORCA, because systematic differences stemming from the choice of basis sets and code are significantly smaller than typical differences between DFT and DLPNO‐CCSD(T) adsorption energies.

**Figure 8:**
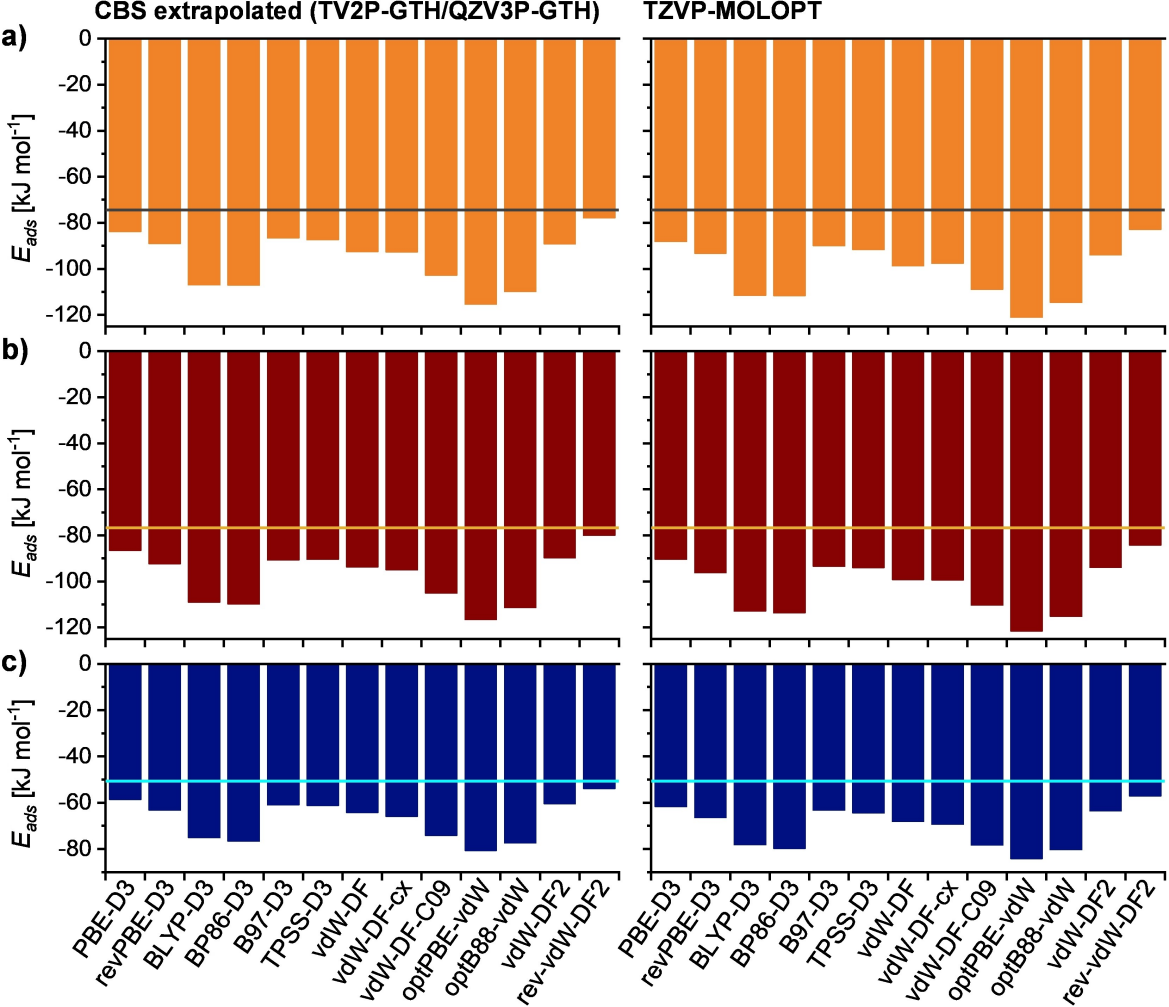
DFT adsorption energies $E_{ads}$ obtained for the three cluster models shown in Figure 7: a) ACA@MOR, Config1, b) ACA@MOR, Config3, c) ACA@FAU. CBS‐extrapolated values (TZV2P‐GTH/QZV3P‐GTH) are shown on the left, values obtained with TZVP‐MOLOPT basis sets on the right. CBS‐extrapolated DLPNO‐CCSD(T) reference values are indicated as horizontal lines: MOR@ACA, Config1: −74.4 kJ mol^−1^, MOR@ACA, Config3: −76.7 kJ mol^−1^, ACA@FAU: −50.6 kJ mol^−1^.

All DLPNO‐CCSD(T) adsorption energies, including those obtained with individual basis sets (with and without correction for basis set superposition error) and CBS‐extrapolated values, are compiled in Table S3.3. A comparison of def2‐SVZ/def2‐TZVP and def2‐TZVPP/def2‐QZVPP extrapolated energies shows that the former are much more negative, by about −14 kJ mol^−1^, indicating that the inclusion of quadruple‐zeta basis sets in the CBS extrapolation is required to obtain reliable results. Figure 8 compares adsorption energies obtained with different dc‐XC functionals in CP2K to the high‐quality CBS‐extrapolated DLPNO‐CCSD(T) values (def2‐TZVPP/def2‐QZVPP) for the three different configurations. On the DFT side, both CBS‐extrapolated adsorption energies based on TZV2P‐/QZV3P‐GTH energies and TZVP‐MOLOPT results are included in the figure.

First of all, it is clearly visible that identical trends across the set of dc‐XC functionals are observed for all three configurations, and for both CBS‐extrapolated and TZVP‐MOLOPT adsorption energies, with TZVP‐MOLOPT delivering systematically more negative values by about −2 to −6 kJ mol^−1^. When comparing the CBS‐extrapolated adsorption energies to the DLPNO‐CCSD(T) reference values, the following observations can be made:

- All dc‐XC functionals have a systematic tendency to deliver too negative adsorption energies, with the mean of signed errors (MSE ) ranging from −3.4 kJ mol^−1^ for rev‐vdW‐DF2 to −37 kJ mol^−1^ for optPBE‐vdW. As rev‐vdW‐DF2 was consistently identified as the functional delivering the least negative adsorption energies, it emerges as approach that provides best agreement with the DLPNO‐CCSD(T) benchmark.
- Among the functionals including a D3 dispersion correction, PBE‐D3 gives best agreement with the benchmark values (MSE =−9.1 kJ mol^−1^), with B97‐D3 and TPSS‐D3 performing slightly worse. These functionals provide errors of similar magnitude as vdW‐DF2 (MSE =−12.6 kJ mol^−1^), the second‐best non‐local approach behind rev‐vdW‐DF2.
- BYLP‐D3, BP86‐D3, vdW‐DF‐C09, optPBE‐vdW, and optB88‐vdW result in very pronounced overbinding with respect to the benchmark, with MSE values exceeding ‐25 kJ mol^−1^.

Although some caution has to be exercised in the interpretation of these results, as will be discussed in more detail below, it is obvious that the observed trends are systematic, and that the qualitative and quantitative differences among different functionals are considerable.

## Discussion

### Effect of basis set size and optimisation strategy

The results obtained in **Parts 1** and **2** show very clearly that the use of DZVP‐SR basis sets results in systematically more negative adsorption energies compared to TZVP‐MOLOPT basis sets. The differences among results obtained with basis sets of different size vary considerably, depending on the dc‐XC functional used and the adsorbate. Reassuringly, the comparison of adsorption energies obtained with TZVP‐MOLOPT basis sets for cluster models to CBS‐extrapolated values (TZV2P‐GTH/QZV3P‐GTH) showed only modest quantitative differences (MSE on the order of −3 to −5 kJ mol^−1^, corresponding to relative errors of 3.5 to 6 %, see bottom of Table S3.1). This indicates that TZVP‐MOLOPT basis sets may constitute a good balance between accuracy and computational cost, especially for periodic systems.

The comparison of results from **Part 1**, where the structures were optimised using DZVP‐SR basis sets, and **Part 2**, where a full optimisation using TZVP basis sets was performed, showed that the optimisation with the larger basis sets had only a marginal influence on the resulting adsorption energies. From the viewpoint of an efficient use of computational resources, it seems thus attractive to routinely combine optimisations with smaller basis sets and adsorption energy calculations using larger basis sets. This strategy could also be employed for AIMD simulations, where a few snapshots from a trajectory might be extracted for a recalculation of energies with larger basis sets. Nevertheless, it is clear that there may also be cases where a full optimisation with triple‐zeta basis sets can be desirable, *e. g*., when aiming at a detailed analysis of the equilibrium structure (generally, use of basis sets beyond triple‐zeta quality for DFT structure optimisations is rarely necessary^[112]^).

Finally, it was shown in **Part 3** that the trends among the different functionals are retained when moving from periodic calculations, in which the positions of the guest molecules were fully optimised, to single‐point calculations using frozen cluster models cut out from PBE‐D3 optimised structures. Given the rather large variations in the equilibrium positions of the adsorbates observed in **Part 2**, at least for some cases, this relative “robustness” of the trends might appear rather surprising. First, it can be inferred that it may be sufficient to pre‐optimise the structures with one functional prior to calculations of the adsorption energies using a larger number of functionals in order to get a first overview. In this way, the set of approaches could be narrowed down to the most promising ones, avoiding computationally demanding optimisations with dc‐XC functionals exhibiting systematic errors. Second, the analysis confirms that clusters can serve as useful model systems to benchmark DFT approaches against higher‐level calculations, for which periodic calculations may be computationally very expensive or even completely unfeasible.

### Comparing different dc‐XC functionals: Quantitative differences, qualitative similarities

All three parts of this work corroborated a massive dependence of the adsorption energies on the choice of dc‐XC functional, with quantitative deviations frequently amounting to several 10 per cent in relative terms. These variations are essentially independent of the choice of basis sets or the optimisation strategy. Hence, it is clear that any study that is aimed at an at least semi‐quantitative prediction of adsorption energies for organic contaminants (or other sizeable organic molecules) in zeolites will have to pay considerable attention to the use of an appropriate DFT approach. On the other hand, the results of **Part 1** showed that qualitative trends are not strongly affected by the dc‐XC functional. Therefore, DFT‐based investigations that are primarily aimed at the identification of zeolite‐guest combinations exhibiting strong interaction should be fairly insensitive to the choice of approach. Although the occurrence of some outliers in Figure 2 serves as a reminder that caution must be exercised in individual cases, the overall findings indicate that the identification of zeolite adsorbents having a high affinity towards a given contaminant should, in the majority of cases, not depend strongly on the dc‐XC functional, at least among the 13 functionals tested.

Compared to the calculation of adsorption energies for different combinations of zeolites and contaminants, the energetic ordering of different adsorption configurations for the same zeolite‐contaminant combination constitutes a more intricate problem. The energy differences among different configurations are often on the order of a few kJ mol^−1^, and it is clear that none of the dispersion‐corrected DFT approaches considered could deliver an accuracy that would allow to determine the energetic ordering with certainty. For those configurations that are not too close in energy, the majority of functionals give qualitatively and quantitatively similar results. It is noteworthy that there is no one‐to‐one correspondence between absolute adsorption energies and relative energies of different configurations: For example, optPBE‐vdW delivers adsorption energies that are 40 to 70 kJ mol^−1^ more negative than those obtained with PBE‐D3 for ACA, IBU, and TCS adsorbed in MOR, but the relative energies of different configurations computed with these two functionals agree to within 1 kJ mol^−1^.

The comparison of the adsorption complexes optimised with different dc‐XC functionals showed that numerous functionals give fairly similar results. Specifically, three of the vdW‐DF1 approaches and both vdW‐DF2 approaches delivered atomic positions that are very close to those obtained with PBE‐D3. The agreement in the coordinates is rather striking when considering that the adsorption energies computed with these methods vary considerably: For example, the smallest deviation in coordinates from PBE‐D3 was found for optPBE‐vdW, which, as discussed in the preceding paragraph, gives much more negative adsorption energies. Rather surprisingly, substantial deviations were apparent among the GGA+D3 functionals, despite the consistent derivation of the dispersion correction term. This indicates that the exchange‐correlation contribution has an important impact on the equilibrium position of the guest molecule, despite the dominant influence of dispersion interactions on the total interaction strength. Altogether, it can be concluded that functionals giving similar adsorption energies do not necessarily deliver similar equilibrium structures, and vice versa. More comprehensive benchmarking studies against higher‐level data should therefore take both interaction energies and structural properties into account.

### Choosing a suitable DFT approach

Although the results have shown that qualitative predictions of trends in adsorption energies will not be heavily affected by the choice of dc‐XC functional, it is clear that one will usually strive to employ an approach that gives reasonably “accurate” absolute values. In this context, the term “accurate” is not meant to reflect the “chemical accuracy” that is often the goal in high‐level benchmarking studies, but to reflect a performance that is largely free from systematic over‐ or underestimations. Compared to gas‐phase adsorption, where sophisticated approaches to derive adsorption energies from experimentally measured adsorption enthalpies have been developed,^[56]^ the situation is more complex for species adsorbed from the liquid phase, and a direct comparison to experimental values does not, at present, appear as a feasible strategy to gauge the performance of DFT approaches. To obtain higher‐level reference values, DLPNO‐CCSD(T) calculations were carried out for a few cluster models. Clearly, this benchmarking remained preliminary for various reasons: First, only one organic molecule and only a few configurations of that molecule were considered in the coupled cluster calculations; future work should include a larger set of species and configurations. Second, the use of cluster models means that the contribution of long‐range interactions is absent both in the benchmark values and in the DFT adsorption energies. If some dc‐XC functionals exhibited systematic errors in the description of these longer‐ranger interactions, their magnitude could not be determined in non‐periodic calculations. Third, it is clear that CBS extrapolation had a major impact on the benchmark DLPNO‐CCSD(T) energies. Although a lot of care was taken in the choice of basis sets and extrapolation strategies to ensure that DFT and DLPNO‐CCSD(T) adsorption energies are directly comparable, future work should explore this in more detail.

Having noted these caveats, it appears reasonable to rank the dc‐XC functionals in terms of their agreement with the DLPNO‐CCSD(T) results. Several of these functionals deliver much more negative adsorption energies than the coupled cluster calculations, agreeing with the previously established trend that dispersion‐corrected DFT approaches tend to overestimate the interaction strength in adsorption complexes. On this basis, BLYP‐D3, BP86‐D3, vdW‐DF‐C09, optPBE‐vdW, and optB88‐vdW can certainly be discarded. While the relative deviations found for vdW‐DF and vdW‐DF‐cx are smaller, they are large enough to rule them out as well. revPBE‐D3, B97‐D3, TPSS‐D3 and vdW‐DF2 exhibit errors on the order of −12 to −14 kJ mol^−1^, better than the previously mentioned functionals, but worse than PBE‐D3, for which the MSE amounts to −9 kJ mol^−1^. The results for PBE‐D3, one of the most widely used dc‐XC functionals in adsorption investigations, are essentially in line with earlier studies of smaller molecules in zeolites, where PBE‐D3 was found to overestimate the interaction strength systematically, but not as prominently as many functionals from the non‐local vdW‐DF1 family.[^36^, ^38^, ^39^, ^40^, ^41^, ^42^, ^43^] Since the CBS‐extrapolated DLPNO‐CCSD(T) adsorption energies are consistently less negative than the DFT values, regardless of the choice of functional, the rev‐vdW‐DF2 functional that was previously found to predict the weakest interaction strength provides the best agreement with the benchmark values, with a mean of signed errors of −3.4 kJ mol^−1^. Even when using TZVP‐MOLOPT basis sets in a routine fashion, without CBS extrapolation, the agreement for rev‐vdW‐DF2 (MSE =−7.5 kJ mol^−1^) remains better than for the second‐best functional (PBE‐D3) with CBS extrapolation. Among the tested set of dc‐XC functionals, rev‐vdW‐DF2 thus emerges as the most attractive choice when aiming to compute reasonably accurate adsorption energies (of course, this does not rule out that other functionals that were not tested here might perform even better). Although there are a few studies in which this functional was used to compute adsorption energies for different guest species in zeolites,[^32^, ^33^, ^113^] these reported no benchmark values from experiment or higher‐level calculations. Broadening the view to adsorption in other materials, Vlaisavljevich et al. used rev‐vdW‐DF2 – together with several other functionals – in a study of CO_2_, CH_4_, and H_2_O adsorption in metal‐organic frameworks (MOFs) with open metal sites.^[114]^ They observed excellent agreement with experimental $E_{ads}$ values (derived from adsorption enthalpies) and metal‐guest equilibrium distances (obtained from in‐situ neutron diffraction). The good performance for MOFs with positively polarised open metal sites could imply that rev‐vdW‐DF2 is also suitable for adsorption in cationic zeolites, where a reasonable representation of the interaction with the cations will be crucial. A good performance of this functional was also observed in DFT studies addressing the adsorption of large aromatic hydrocarbons on coinage metals,^[115]^ of H_2_ on graphene,^[116]^ of water on carbon nanostructures,[^117^, ^118^] and of benzene on gold and acetylene on sodium chloride.^[119]^

Finally, it is worth pointing out that the coordinates of the zeolite framework atoms were held fixed in all calculations presented here. While it is acknowledged that a full relaxation of the framework should be included in an accurate description of adsorption phenomena, it is not expected that it would result in qualitatively different findings across the set of functionals studied. As a case in point, the “deformation energy” (the change in total energy of the deformed zeolite framework in the adsorption complex with respect to the optimised guest‐free state) mostly remained well below 10 kJ mol^‐1^ (and never exceeded 12 kJ mol^−1^) in recent studies of CBZ and TCS adsorption in all‐silica zeolites.[^32^, ^33^] Increased deformation energies for protonated zeolites show that the relaxation of the framework atoms becomes more important in studies of systems containing a negatively charged framework and charge‐balancing species. The rev‐vdW‐DF2 functional was also found to provide good agreement with experiment when considering structural parameters of all‐silica zeolites and AlPO_4_ zeotypes and (where available) relative stabilities of all‐silica zeolites with respect to α‐quartz,^[70]^ indicating that its usage should not result in pronounced systematic errors in the description of the zeolite structure. The relative robustness of this functional was corroborated for different groups of weakly bound solids in a wide‐ranging benchmarking study.^[79]^

## Concluding Remarks

Calculations for a set of 21 organic contaminants in two all‐silica zeolites that employed 13 dispersion‐corrected DFT approaches revealed significant quantitative differences in the computed adsorption energies. Qualitative trends, however, were not so heavily affected, indicating that different dc‐XC functionals should give similar answers when attempting to identify zeolites having a high affinity towards a given contaminant. Even when comparing different configurations of one molecule in the same zeolite, several functionals gave very similar results, both in terms of relative energies and equilibrium positions of the adsorbed organics. A benchmarking of adsorption energies against high‐level DLPNO‐CCSD(T) calculations, performed for acetaminophen interacting with clusters cut out from the zeolite structures, showed that all considered dc‐XC functionals deliver more negative adsorption energies than the reference method. Among them, rev‐vdW‐DF2 performs best, with only a very modest tendency towards overbinding, thus appearing as a suitable choice for future DFT studies of organic contaminants or related functional organic molecules in all‐silica zeolites. While being computationally slightly more demanding than, for example, PBE‐D3 (with SCF iterations for identical systems taking roughly 40 % longer in CP2K calculations), this functional is still applicable to zeolites with large unit cells, where the use of more demanding approaches (*e. g*., hybrid functionals) would incur a significant computational overhead. Calculations using rev‐vdW‐DF2 should also provide a useful starting point for the validation of FF parameters, which could then, in turn, be employed in MC or MD simulations to investigate the adsorption and diffusion of large organics in zeolites.

Clearly, the present study could be expanded in various directions. First of all, efforts should be undertaken to obtain a larger set of benchmark energy values against which the DFT results can be compared more comprehensively, either using high‐level calculations or experiments. For the set of dc‐XC functionals studied here, the already identified trends could provide some indications for future studies, potentially allowing to discard some of the functionals and consider other, more promising ones instead. Second, further work should include temperature effects, and potentially also consider the choice of an appropriate reference state that would ultimately allow the prediction of adsorption enthalpies for species adsorbed from aqueous solution. Third, adsorption‐induced deformations of the zeolite framework should be accounted for. Finally, protonated or cation‐exchanged zeolites can be more attractive than all‐silica zeolites for some applications involving the adsorption of functional organic molecules. Future DFT studies of these systems, which have a more heterogeneous charge distribution, would require further validation. As it is well known that the PBE‐D3 functional exhibits systematic errors for cationic zeolites,^[40]^ it would be very interesting to evaluate whether rev‐vdW‐DF2 performs better for these materials.

## Supporting Information

EXCEL files S1.xlsx, S2.xlsx and S3.xlsx compile the results of **Part 1**, **2**, and **3**. The optimised structures from **Part 1** and **2** are included in ZIP archives S1_Contaminants.zip, S1_MOR.zip, S1_FAU.zip, and S2_MOR_Configs.zip. The cluster models used in **Part 3** as well as output files of ORCA calculations are included in the archive S3_CCSD_T_ORCA.zip. Sample inputs for CP2K calculations are provided in the archive Sample_inputs_CP2K.zip.

## Conflict of interests

The authors declare no conflict of interest.

1

## Data Availability

The data that support the findings of this study are available in the supplementary material of this article.
